# Assessing the quality of life in patients with drug-resistant tuberculosis: a cross-sectional study

**DOI:** 10.1186/s12890-024-03119-1

**Published:** 2024-06-27

**Authors:** He Wang, Jiayi Gu, Lijun Zhang, Yan Song

**Affiliations:** 1 School of Nursing, Nanjing University of Chinese Medicine, No.138 Xianlin Avenue, Qixia District, Nanjing, Jiangsu Province P.R. China; 2 Department of Tuberculosis, The Second Hospital of Nanjing, Affiliated to Nanjing University of Chinese Medicine, No.1 Rehabilitation Road, Tangshan Street, Jiangning District, Nanjing, Jiangsu Province P.R. China; 3 Department of Nursing, The Second Hospital of Nanjing, Affiliated to Nanjing University of Chinese Medicine, No.1-1 Zhongfu Road, Gulou District, Nanjing, 210003 Jiangsu P.R. China

**Keywords:** Drug resistance, Drug-resistant tuberculosis, Quality of life, WHOQOL-BREF, Cross-sectional studies

## Abstract

**Background:**

This study investigated the current status of the quality of life (QOL) of drug-resistant tuberculosis (DR-TB) patients in Nanjing, China, and analyzed the influencing factors.

**Methods:**

The survey was conducted among patients with DR-TB who were hospitalized in the tuberculosis department of the Second Hospital of Nanjing (Nanjing Public Health Medical Center) from July 2022 to May 2023. The Chinese version of the World Health Organization Quality of Life (WHOQOL-BREF) questionnaire was used to investigate the QOL levels of patients with DR-TB, and a multiple linear regression model was used to analyze the QOL influencing factors.

**Results:**

A total of 135 patients participated in the study; 69.6% were male, the average age was 46.30 ± 17.98 years, 13.33% had an education level of elementary school or below, and 75.56% were married. The QOL scores were 51.35 ± 17.24, 47.04 ± 20.28, 43.89 ± 17.96, and 35.00 ± 11.57 in the physiological, psychological, social, and environmental domains, respectively. The differences between the four domain scores and the Chinese normative results were statistically significant (*P* < 0.05). The results of multiple linear regression analysis showed that the factors related to the physiological domain included residence, family per-capita monthly income, payment method, adverse drug reactions (ADRs), and comorbidities; psychological domain correlates included educational level, family per-capita monthly income, course of the disease, and caregivers; social domain correlates included age and comorbidities; and factors related to the environmental domain included age, education level, and comorbidities.

**Conclusions:**

In Nanjing, China, patients with younger age, higher education level, living in urban areas, high family per-capita monthly income, no adverse drug reactions, no comorbidities, and having caregivers have better quality of life. Future interventions to improve the quality of life of patients with drug-resistant tuberculosis could be tailored to a specific factor.

**Supplementary Information:**

The online version contains supplementary material available at 10.1186/s12890-024-03119-1.

## Background

Drug-resistant tuberculosis (DR-TB) is a type of tuberculosis in which a patient is infected with Mycobacterium tuberculosis (MTB) that has been tested and proven to be resistant to one or more anti-TB drugs [1]. The 2023 WHO global TB report showed that an estimated 410,000 patients globally developed multidrug resistant or rifampicin resistant TB (MDR/RR-TB) by 2022, an estimate that is lower than the 450,000 and 437,000 in 2021 and 2020, respectively; however, only 175,650 patients with MDR/RR-TB were confirmed and enrolled in standardized treatment, or only 43% of the MDR/RR-TB patients were diagnosed and enrolled in treatment, which is still lower than the pre-pandemic 2019 data (181,533) [2]. In addition, the estimated number of MDR/RR-TB cases in China is 30,000, which has been on a downward trend since 2015, but the treatment success rate of MDR/RR-TB cases registered in 2020 and initiated on treatment with a second-line anti-TB drug regimen (2-year cycle, with regression evaluated in 2022) is 51%, which is significantly lower than the global average in 2020 (63%) [2]. Notably, DR-TB is a serious public health problem worldwide with high morbidity, substantial mortality, long treatment cycles, and prohibitively expensive treatment [3–5]. Additionally, it has long-term and serious risks to the overall health of individuals and is associated with poor quality of life (QOL) [6]. Living with DR-TB puts individuals at a wide variety of negative outcomes, including mental health, psychological wellbeing, physical condition, social relations and socioeconomic wellbeing [4, 7, 8]. Therefore, the management of DR-TB should receive widespread attention from researchers.

An individual’s perception of their current situation in life, as well as their expectations, goals, and standards, according to the WHO, is referred to as their QOL [9]. In clinical practice, QOL provides a comprehensive and accurate picture of patients’ psychological status, social functioning, treatment outcome and recovery status [10]. Self-reported QOL is an important aid in understanding and quantifying the actual impact of DR-TB on patients, and regular assessment of QOL not only helps to assess the effectiveness of patient treatment but also provides a basis for timely and appropriate interventions by health care providers [11–13]. A Peruvian study revealed that low QOL scores at the start of TB treatment were associated with death, adverse treatment outcomes, and treatment non-completion, and that patient QOL fully recovered after successful completion of treatment. In addition, the study emphasized that the impairment of QOL associated with TB defines important multisystem ill health, and the use of QOL evaluation tools may have a role in helping to identify who should be offered enhanced care aiming to reduce the risk of mortality [14]. Hence, the QOL of individuals living with DR-TB has been considered an important outcome indicator for healthcare decision-making and intervention effect evaluation.

Research on the QOL of people with drug-resistant TB is now being conducted in a number of LMICs, including Vietnam [15], India [12, 16], Eritrea [4], and Yemen [17]. A cross-sectional study in Vietnam used the stigma scale, depression scale and health-related quality of life (HRQOL) scale investigate drug-sensitive tuberculosis (DS-TB) patients and multidrug-resistant tuberculosis (MDR-TB) patients. When the differences between the two populations were compared, it was found that MDR-TB patients had a greater incidence of depression than did DS-TB patients, MDR-TB patients had greater mean depression and stigma scores than did DS-TB patients, and MDR-TB patients had a lower HRQOL than did DS-TB patients [15]. A study in Eritrea using the WHOQOL-BREF scale yielded similar results, with RR/MDR-TB patients having a worse HRQOL than DS-TB patients [4]. A prospective cohort study in Yemen focused on HRQOL during and after treatment in multidrug-resistant tuberculosis (MDR-TB) patients and identified risk factors that predicted differences in HRQOL scores [17]. The MDR-TB patients completed the SF-36 V2 survey at the beginning and end of treatment and after 1 year of follow up. The results of the study showed an increase in scores on all domains of the HRQOL at the completion of treatment compared to the beginning of treatment, but a decrease in the results of the survey was observed at 1 year of follow up after treatment. Age, smoking status, history of streptomycin use, baseline lung cavity, stigma, residence, marital status and length of sickness before MDR-TB diagnosis were predictive of score differences [17]. Some studies have suggested that adverse events (AE) in the first few months of treatment may be responsible for further reductions in HRQOL [18]. Furthermore, in a systematic review, a limited number of QOL and health-related QOL studies have been conducted in patients with MDR-TB and XDR-TB, especially in patients with XDR-TB. A large number of MDR-TB and XDR-TB patients continue to have sequelae after completing treatment, reducing the health-related QOL of these individuals [19].

However, in China, the management of DR-TB patients has focused mainly on the research and development of new drugs and diagnostic methods, and relatively few studies have been conducted to assess the QOL of DR-TB patients. The two most well-known QOL instruments are the Short Form 36-item Health Survey (SF-36) and the World Health Organization Quality of Life-BREF (WHOQOL-BREF) [20]. Most previous studies have used the SF-36 to assess the QOL of patients, but the SF-36 offers little information to help researchers understand the unique experiences among TB patients [17, 20, 21]. Huang et al. [22] concluded, “The SF-36 and WHOQOL-BREF appear to measure different constructs: the SF-36 measures health-related QOL, while the WHOQOL-BREF measures global QOL”. Consequently, this study selected the WHOQOL-BREF to assess DR-TB patients’ global QOL and explored the associated factors associated with QOL to provide a reference for developing programs to improve and enhance the QOL of DR-TB patients.

## Methodology

### Study design

This study employed a cross-sectional research design to assess DR-TB patients’ global QOL and explore the associated factors affecting QOL.

### Participants and data collection

A convenience sampling method was used to recruit patients with DR-TB who were hospitalized in the tuberculosis department of the Second Hospital of Nanjing (Nanjing Public Health Medical Center) from July 2022 to May 2023 as the study population. The target subjects of this study met the following criteria: (1) met the diagnostic criteria in the Expert Consensus on Drug Resistance Testing of MTB published in 2019 in China, and were diagnosed with DR-TB infection confirmed by MTB culture and drug susceptibility testing, as well as MTB and Rifampicin Resistance Detection Technique (Xpert MTB/RIF). The following criteria were met for DR-TB patients: mono-drug resistant tuberculosis (MR-TB), multidrug-resistant tuberculosis (MDR-TB), multidrug-resistant tuberculosis (PDR-TB), and extensively drug-resistant tuberculosis (XDR-TB); (2) aged ≥ 18 years; (3) agreed to participate in the survey and were able to fill out the questionnaire independently or with the help of the investigator; and (4) received treatment for DR-TB recommended in the Guidelines for Chemotherapy of DR-TB published in 2019. Those with disturbance of consciousness, speech disorders, comprehension disorders or other serious diseases (such as malignant tumors) were excluded. After removing incomplete questionnaires, we finally obtained an effective sample of 135 patients.

After unified training, the investigators selected patients with DR-TB who were hospitalized in the tuberculosis unit for the survey according to the inclusion and exclusion criteria. Before conducting the survey, we explained its purpose and obtained informed consent from the patients and their families. Then, the investigators distributed the questionnaires. It took approximately 15 min to ensure that every selected patient completed an anonymous questionnaire independently. All questionnaires were collected on the spot, and the results of the survey improved over time.

### Measures

#### General characteristics

The participants provided information regarding their gender, age, education level, marital status, residence, occupation, family per-capita monthly income, payment method, BMI, course of disease, drug-resistant types, retreatment, ADRs, comorbidities, and caregivers.

#### WHOQOL-BREF questionnaire

The WHOQOL-BREF questionnaire, a brief version of the WHOQOL-100, was used to assess the subjects’ QOL in the last four weeks [23]. It consisted of 26 items. Item one and item two separately assessed the overall perception of QOL and satisfaction with health, while the other 24 items were classified into four domains: physiological, psychological, social, and environmental [24]. The equation recommended by the WHO is used to calculate the total score of each domain [25]. Because the number of items varies across the four domains, the score of each domain is calculated by multiplying the average score of all items in the domain by the same factor of 4, and the percentage conversion method for each domain is (each domain score-4) × (100/16). Each item was rated on a 5-point Likert scale from 1 to 5, and the total score of each domain was between 4 and 20, which was then normalized to a score from 0 to 100. The higher the score is, the better the QOL. We used the Chinese version of the WHOQOL-BREF questionnaire, which has been widely used and validated in China [26]. Additionally, the level of internal consistency for the WHOQOL-BREF questionnaire (all 26 items) measured by Cronbach’s alpha coefficient was 0.87 for this study. For the physiological domain, it was 0.73, 0.75 for the psychological domain, 0.72 for the social domain, and 0.83 for the environmental domain, indicating that the WHOQOL-BREF questionnaire has adequate reliability.

### Data analysis

All the statistical analyses were performed using SPSS for Windows (version 23.0; SPSS Inc., Chicago, IL, USA). Descriptive statistics were used to describe the characteristics of the participants. Categorical and numerical variables are presented as the number (%) and mean (standard deviation; SD), respectively. T tests were used to compare the results with the national norm among China’s general population [27] and to assess differences between the means of individual variables and differences in the mean scores of various domains of the WHOQOL-BREF. Differences between ≥ 3 groups were analyzed by analysis of variance (ANOVA). Furthermore, we developed a multivariate linear regression model, with significant social demographic information and clinical factors of DR-TB patients (*P* < 0.05) as independent variables and the QOL scores for each domain as dependent variables, to assess the relationships between QOL and social demographic and clinical factors. All hypothesis testing was based on 2-sided tests with an alpha level of 0.05. Detailed coding information of the variables is presented in Table S1.

## Results

### General characteristics of the participants

The detailed sociodemographic and clinical characteristics of the participants are displayed in Table 1. Of the 135 DR-TB patients, 94 (69.6%) were males and 41 (30.4%) were females. The participants’ ages ranged from 18 to 85, with the age group below 40 accounting for 30.4%. A total of 117 (86.67%) participants had received education above junior high school, and 106 (78.52%) had marital experience. The majority of families (65.19%) came from rural areas, more than 80.0% of participants had jobs, and most participants perceived their family economic status to be general or above. Additionally, only 17.78% had to pay out-of-pocket for treatment. Based on the BMI cutoffs, more than 50.0% of participants had a normal BMI. Additionally, Table 1 shows that 99 participants (73.33%) reported a longer duration of DR-TB (> 3 months). According to the clinical characteristics of DR-TB, the prevalence rates of mono-drug resistant tuberculosis (MR-TB), multidrug resistant tuberculosis (MDR-TB), polydrug resistant tuberculosis (PDR-TB), and extensively drug-resistant tuberculosis (XDR-TB) were 25.2%, 8.9%, 62.2%, and 3.7%, respectively, in this sample. The table also reveals that a total of 49.6% of participants experienced DR-TB retreatment, and 24.4% of participants had comorbidities. Notably, more than half of the participants (62.2%) experienced ADRs, and most participants (71.9%) had no caregivers.

**Table 1:** Sociodemographic and clinical characteristics of DR-TB patients (*N* = 135)

Variables		*n*	(%)
Gender	Male	94	69.6
	Female	41	30.4
Age (year)	< 40 years old	54	40.0
	40–60 years old	44	32.6
	> 60 years old	37	27.4
Education level	Primary school or less	18	13.33
	Junior High School	42	31.11
	Senior School or Technical Secondary School	39	28.89
	University or above	36	26.67
Marital Status	Married	102	75.56
	Single	29	21.48
	Divorced or widowed	4	2.96
Residence	Rural	88	65.19
	Urban	47	34.81
Occupation	Unemployed	25	18.52
	Farmer or worker	36	26.67
	Staff	32	23.70
	Students	14	10.37
	Self-employed	5	3.70
	Retirement	23	17.04
Family per-capita monthly income (RMB)	< 1500	22	16.30
	1500–3000	50	37.04
	3000–5000	44	32.59
	> 5000	19	14.07
Payment method	Self-pay	24	17.78
	Provincial or municipal health insurance	40	29.63
	New Rural Cooperative Medical Care	71	52.59
BMI	< 18.5 kg/m^2^	8	5.93
	18.5–22.9 kg/m^2^	96	71.11
	≥ 23.0 kg/m^2^	31	22.96
Course of disease	< 3 months	36	26.67
	3–6 months	37	27.41
	6–12 months	50	37.04
	> 12 months	12	8.88
Drug-resistant types	MR-TB	34	25.19
	PDR-TB	12	8.88
	MDR-TB	84	62.22
	XDR-TB	5	3.70
Retreatment	No	68	50.37
	Yes	67	49.63
ADRs	No	51	37.78
	Yes	84	62.22
Comorbidities	No	102	75.56
	Yes	33	24.44
Caregivers	No	97	71.85
	Yes	38	28.15

Note. MR-TB, Mono-drug resistance tuberculosis; PDR-TB, Polydrug resistant tuberculosis; MDR-TB, Multi-drug resistance tuberculosis; XDR-TB, Extensively drug-resistant tuberculosis; ADRs, Adverse drug reactions

### Comparison of QOL between our study population and China’s general population

As shown in Table 2, the scores of the physiological, psychological, social, and environmental domains were 51.35 ± 17.24, 47.04 ± 20.28, 43.89 ± 17.96, and 35.00 ± 11.57, respectively. The QOL scores in all domains were significantly different from those of China’s general population (*P* < 0.001). Of the four domains, the score in the physiological domain ranked the highest and that in the environmental domain ranked the lowest.

**Table 2:** QOL’s comparison between DR-TB patients in Nanjing and domestic general population (Mean ± *SD*)

Fields	DR-TB patients	China Standing Model	t-value	*P*-value
Physiological	51.35 ± 17.24	73.97 ± 13.84	-15.247	< 0.001
Psychological	47.04 ± 20.28	66.65 ± 14.21	-11.231	< 0.001
Social	43.89 ± 17.96	65.76 ± 14.08	-14.149	< 0.001
Environmental	35.00 ± 11.57	56.59 ± 15.15	-21.685	< 0.001

### QOL of DR-TB patients

There were statistically significant differences in the social and environmental domain scores by gender (*P* < 0.05), with females having higher scores of 50.20 ± 16.92 and 38.34 ± 11.94, respectively. Except for the QOL score in the psychological domain, the scores in the other three domains were significantly different in terms of age (*P* < 0.05). There were significant differences in scores among the four education level groups (*P* < 0.05), with those with less than primary education having the worst scores in all QOL domains. Those who were single had significantly better scores in the social and environmental domains than those with other marital statuses (*P* < 0.05). Additionally, participants’ residence, payment method, and BMI differed significantly only in the physiological domain score (*P* < 0.05). The scores in the physiological and social domains were significantly different among different occupations (*P* < 0.05). Those with lower family per capita monthly income had lower scores in the physiological and psychological domains (*P* < 0.05). Furthermore, the longer the duration of the disease, the lower the QOL score in the physiological, psychological, and environmental domains (*P* < 0.05). There were significant differences in scores in physiological and psychological domains between those with and without ADRs, with ADRs receiving lower scores than those without ADRs (*P* < 0.05). Except for the psychological domain, the scores in the other three domains were significantly different, with those who had comorbidities having relatively lower scores than those without comorbidities (*P* < 0.05). Moreover, patients with caregivers had relatively higher scores in all domains than did those without caregivers (*P* < 0.05). A comparison of the QOL scores according to sociodemographic, clinical and disease-related characteristics is presented in Table 3.

**Table 3:** Comparison of QOL scores by domains according to socio-demographic factors (*N* = 135)

Variables		Physiological domain	Psychological domain	Social domain	Environmental domain
Gender	Male (*n* = 94)	51.82 ± 17.07	47.44 ± 19.91	41.13 ± 17.79	33.55 ± 11.15
	Female (*n* = 41)	50.26 ± 17.79	46.14 ± 21.33	50.20 ± 16.92	38.34 ± 11.94
	*t*-value	0.48	0.34	2.76	2.45
	*P*-value	0.630	0.734	**0.007**	**0.026**
Age (year)	< 40 years old (*n* = 54)	48.28 ± 17.64	50.39 ± 21.28	58.33 ± 12.64	40.63 ± 9.48
	40–60 years old (*n* = 44)	58.20 ± 16.62	45.64 ± 20.49	41.67 ± 11.65	31.25 ± 8.74
	> 60 years old (*n* = 37)	47.68 ± 15.25	43.82 ± 18.25	25.45 ± 11.61	31.26 ± 13.98
	*F*-value	5.51	1.31	82.89	14.68
	*P*-value	**0.005**	0.273	**< 0.001**	**< 0.001**
Education level	Primary school or less (*n* = 18)	38.10 ± 12.13	30.35 ± 14.44	30.09 ± 14.61	25.35 ± 11.03
	Junior High School (*n* = 42)	55.02 ± 16.79	47.52 ± 20.37	37.90 ± 14.04	33.26 ± 10.37
	Senior School or Technical Secondary School (*n* = 39)	53.21 ± 16.46	50.21 ± 19.63	44.87 ± 20.20	36.62 ± 12.80
	University or above (*n* = 36)	51.69 ± 18.20	51.39 ± 19.87	55.71 ± 12.09	40.11 ± 8.23
	*F*-value	4.70	5.43	20.62	8.24
	*P*-value	**0.004**	**0.001**	**< 0.001**	**< 0.001**
Marital Status	Married (*n* = 120)	51.50 ± 16.97	46.41 ± 20.02	40.69 ± 17.41	33.70 ± 11.85
	Single (*n* = 29)	51.35 ± 18.49	49.28 ± 22.19	58.05 ± 11.24	40.41 ± 8.43
	Divorced or widowed (*n* = 4)	47.32 ± 18.98	46.88 ± 14.98	22.92 ± 12.50	28.91 ± 14.06
	*F*-value	0.112	0.22	26.02	4.61
	*P*-value	0.894	0.800	**< 0.001**	**0.012**
Residence	Rural (*n* = 88)	47.93 ± 17.34	46.12 ± 20.87	43.94 ± 18.21	34.13 ± 12.11
	Urban (*n* = 47)	57.75 ± 15.27	48.76 ± 19.23	43.79 ± 17.67	36.64 ± 10.40
	*t*-value	1.03	0.35	0.092	0.74
	*P*-value	**0.001**	0.474	0.965	0.231
Occupation	Unemployed (*n* = 25)	40.29 ± 17.93	37.00 ± 21.76	41.99 ± 19.02	32.00 ± 13.60
	Farmer or worker (*n* = 36)	56.05 ± 15.92	50.58 ± 18.48	43.06 ± 19.63	35.42 ± 11.86
	Staff (*n* = 32)	53.01 ± 16.75	49.22 ± 21.49	49.74 ± 15.33	35.45 ± 10.49
	Students (*n* = 14)	53.83 ± 15.50	48.51 ± 19.92	59.52 ± 10.77	41.52 ± 8.08
	Self-employed (*n* = 5)	59.29 ± 17.79	50.83 ± 24.54	50.00 ± 8.33	35.63 ± 5.68
	Retirement (*n* = 23)	50.47 ± 16.20	47.67 ± 17.43	28.26 ± 11.44	32.88 ± 12.13
	*F*-value	3.18	1.61	8.21	1.43
	*P*-value	**0.010**	0.162	**< 0.001**	0.220
Family per-capita monthly income (RMB)	< 1500 (*n* = 22)	41.56 ± 18.20	35.82 ± 19.40	40.15 ± 21.46	32.11 ± 16.34
	1500–3000 (*n* = 50)	52.71 ± 15.78	49.08 ± 21.28	42.67 ± 18.64	34.94 ± 11.59
	3000–5000 (*n* = 44)	52.92 ± 18.59	49.43 ± 18.84	44.51 ± 17.14	35.66 ± 9.98
	> 5000 (*n* = 19)	55.45 ± 13.28	49.12 ± 18.92	50.00 ± 12.42	37.01 ± 8.09
	*F*-value	3.09	2.79	1.15	0.57
	*P*-value	**0.029**	**0.043**	0.332	0.640
Payment method	Self-pay (*n* = 24)	41.81 ± 18.32	41.49 ± 24.22	48.96 ± 19.08	35.55 ± 12.01
	Provincial or municipal health insurance (*n* = 40)	54.82 ± 14.55	48.75 ± 18.35	41.88 ± 15.62	34.61 ± 9.89
	New Rural Cooperative Medical Care (*n* = 71)	52.62 ± 17.35	47.95 ± 19.85	43.31 ± 18.72	35.04 ± 12.41
	*F*-value	4.95	1.11	1.25	0.05
	*P*-value	**0.008**	0.331	0.290	0.952
BMI	< 18.5 kg/m^2^ (*n* = 8)	33.04 ± 9.11	8.70 ± 1.94	8.70 ± 3.14	7.89 ± 1.93
	18.5–22.9 kg/m^2^ (*n* = 96)	53.20 ± 17.30	10.27 ± 1.97	11.42 ± 2.90	10.01 ± 1.71
	< 30 kg/m^2^ (*n* = 31)	50.95 ± 15.99	10.04 ± 2.04	11.26 ± 1.95	9.83 ± 1.36
	*F*-value	4.06	1.77	1.51	1.01
	*P*-value	**0.009**	0.157	0.215	0.389
Course of disease	< 3 months (*n* = 36)	60.71 ± 16.40	58.33 ± 19.13	42.82 ± 18.60	33.86 ± 13.06
	3–6 months (*n* = 37)	42.86 ± 14.51	50.00 ± 16.93	50.23 ± 19.51	40.12 ± 11.57
	6–12 months (*n* = 50)	53.57 ± 16.77	41.67 ± 19.56	41.33 ± 16.06	33.25 ± 9.08
	> 12 month (*n* = 12)	40.18 ± 11.61	26.43 ± 12.77	38.20 ± 14.85	29.95 ± 12.17
	*F*-value	10.25	11.47	2.39	3.68
	*P*-value	**< 0.001**	**< 0.001**	0.072	**0.019**
Drug-resistant types	MR-TB (*n* = 34)	53.26 ± 16.86	45.83 ± 19.76	44.12 ± 18.29	35.30 ± 12.44
	PDR-TB (*n* = 12)	53.27 ± 13.23	46.88 ± 14.56	38.19 ± 10.93	33.34 ± 5.39
	MDR-TB (*n* = 84)	50.17 ± 18.26	46.68 ± 20.71	44.35 ± 18.97	35.16 ± 12.17
	XDR-TB (*n* = 5)	53.57 ± 11.57	61.67 ± 28.17	48.33 ± 10.86	34.38 ± 6.63
	*F*-value	0.34	0.91	0.52	0.29
	*P*-value	0.795	0.436	0.670	0.961
Retreatment	No (*n* = 68)	51.79 ± 17.97	49.14 ± 21.12	46.20 ± 18.07	36.22 ± 11.16
	Yes (*n* = 67)	50.91 ± 16.58	44.91 ± 19.32	41.54 ± 17.68	33.77 ± 11.92
	*t*-value	0.30	1.22	1.51	1.23
	*P*-value	0.768	0.227	0.132	0.221
ADRs	No (*n* = 51)	58.40 ± 17.62	51.96 ± 19.99	44.93 ± 17.17	35.97 ± 11.27
	Yes (*n* = 84)	47.07 ± 15.60	44.05 ± 19.99	43.25 ± 18.50	34.41 ± 11.77
	*t*-value	3.90	2.23	0.53	0.76
	*P*-value	**< 0.001**	**0.028**	0.600	0.450
Comorbidities	No (*n* = 102)	53.61 ± 16.90	48.41 ± 20.16	45.75 ± 16.94	36.40 ± 10.65
	Yes (*n* = 33)	44.37 ± 16.63	42.82 ± 20.39	38.13 ± 19.99	30.68 ± 13.29
	*t*-value	2.74	1.38	2.15	2.52
	*P*-value	**0.007**	0.170	**0.034**	**0.013**
Caregivers	No (*n* = 97)	49.01 ± 16.20	44.55 ± 19.53	41.58 ± 17.28	33.48 ± 10.87
	Yes (*n* = 38)	57.33 ± 18.55	53.40 ± 21.04	49.78 ± 18.53	38.90 ± 12.49
	*t*-value	2.58	2.32	2.43	2.49
	*P*-value	**0.011**	**0.022**	**0.016**	**0.014**

Note. MR-TB, Mono-drug resistance tuberculosis; PDR-TB, Polydrug resistant tuberculosis; MDR-TB, Multi-drug resistance tuberculosis; XDR-TB, Extensively drug-resistant tuberculosis; ADRs, Adverse drug reactions

### Multifactorial analysis of the QOL of patients with DR-TB

We performed multivariate linear regression analysis using the scores of the four domains of QOL as the dependent variables and a series of statistically significant sociodemographic, clinical and disease-related characteristics (age, gender, residence, education level, occupation, family per-capita monthly income, payment method, BMI, course of disease, ADRs, comorbidities, and caregivers) as the independent variables. Table 4 presents the results of the regression analysis. For QOL in the physiological domain, residence (*β* = 0.333), family per-capita monthly income (*β* = 0.193), payment method (*β* = 0.222), ADRs (*β* = -0.282), and comorbidities (*β* = -0.154) were associated factors. For QOL in the psychological domain, education level (*β* = 0.208), family per capita monthly income (*β* = 0.180), course of disease (*β* = -0453), and caregivers (*β* = 0.166) were associated factors. For QOL in the social domain, age (*β* = -0.653) and comorbidities (*β* = -0.145) were associated factors. For QOL in the environmental domain, age (*β* = -0.216), education level (*β* = 0.218), and comorbidities (*β* = -0.172) were associated factors.

**Table 4:** Results of regression analysis of factors influencing QOL in patients with DR-TB (*N* = 135)

Dependent variable	Independent variable	Regression coefficient	Standard error	Standardized regression coefficients (β)	t	*P*
QOL-Physiological domain	Residence	12.021	3.207	0.333	3.748	< 0.001
	Family per-capita monthly income	3.592	1.422	0.193	2.527	0.013
	Payment method	5.005	1.884	0.222	2.657	0.009
	ADRs	-10.000	2.953	-0.282	-3.386	0.001
	Comorbidities	-6.158	2.958	-0.154	-2.082	0.039
QOL-Psychological domain	Education level	4.183	1.486	0.208	2.814	0.006
	Family per-capita monthly income	3.935	1.620	0.180	2.429	0.017
	Course of disease	-9.573	1.720	-0.453	-5.565	< 0.001
	Caregivers	7.455	3.316	0.166	2.248	0.026
QOL-Social domain	Age	-14.394	1.569	-0.653	9.173	< 0.001
	Comorbidities	-6.051	2.334	-0.145	-2.593	0.011
QOL-Environmental domain	Age	-3.067	1.413	-0.216	-2.171	0.032
	Education level	2.489	1.081	0.218	2.303	0.023
	Comorbidities	-4.622	2.101	-0.172	-2.200	0.030

Note. Multiple linear regression model p-value was set at < 0.05. Physiological domain (*F* = 6.179, *P* < 0.001, *R*^2^ = 0.356, adjusted *R*^2^ = 0.298); psychological domain (*F* = 12.024, *P* < 0.001, *R*^2^ = 0.318, adjusted *R*^2^ = 0.291); social domain (*F* = 29.220, *P* < 0.001, *R*^2^ = 0.617, adjusted *R*^2^ = 0.596); and environmental domain (*F* = 6.096, *P* < 0.001, *R*^2^ = 0.251, adjusted *R*^2^ = 0.210). ADRs, Adverse drug reactions

## Discussion

The present study used the WHOQOL-BREF instrument to assess QOL among DR-TB patients in Nanjing and attempted to explore the associated factors. Located in East China, Nanjing is an ancient capital with a relatively developed economy and profound cultural heritage. In Nanjing, studies have shown that the total rate of DR-TB during 2017–2020 was 32.7% [28], lower than that during 2002–2014 (49.53 ± 8.57%) [29] but still higher than that in the areas of Hainan (24.9%) [30], Dalian (29.59%) [31] and Hangzhou (23.82%) [32] in China. At the same time, a meta-analysis also noted out that the prevalence of any drug resistance among new and retreatment cases was significantly higher in eastern China than in other regions of China [33]. Therefore, it is crucial to strengthen attention to populations who live with DR-TB, especially focusing on the QOL of DR-TB patients.

To assess the QOL level of DR-TB patients in Nanjing, we compared the QOL scores of participants with the results of the study conducted among the Chinese general population (8366 participants, 2016). The results showed that our study population differed significantly from the general Chinese population in QOL in all domains and that DR-TB patients had lower mean scores in all domains. This indicates that DR-TB has a serious impact on patients’ QOL, and the phenomenon needs to be brought to the attention of researchers. Furthermore, the domain that appeared to be most affected was the environmental domain, which is consistent with other studies [12]. The lowest average QOL scores of the environmental domain may be due to a poor home environment, inability to participate in recreational/leisure activities, lack of appropriate opportunities to acquire new information and skills, and dissatisfaction with the existing transportation and physical environment (pollution/noise/traffic/climate) [34]. Therefore, future work should strengthen the construction of the environmental domain in the QOL of DR-TB patients.

One study found that better QOL was found among well-educated people [19]. In our study, some significant education level and caregiver differences were found in QOL in all domains. DR-TB patients become socially isolated and lose support networks due to hospitalization, stigma, and difficulties in maintaining family life. Caregivers providing support can help patients interact socially and increase their sense of belonging to an intimate social circle [4]. Our results also showed a significant correlation between the disease course and the QOL of physiological, psychological, and environmental domains (*P* < 0.05). The longer the disease course, the lower the QOL of those domains. Among them, participants with a disease duration of more than 12 months had the lowest QOL. In the Nuwagira et al. [35] study, participants who completed 18 months of pulmonary MDR TB treatment and were considered cured reported lower QOL. The reasons may be due to loss of income due to the disease, debts incurred during treatment, and destruction of the lung parenchyma and interstitium leading to post-TB lung disease [36]. We found that participants with no ADRs reported better QOL in the physiological and psychological domains (*P* < 0.05).

Exploring the QOL scores among DR-TB patients, we found that age had a significant effect on scores in the physiological, psychological and environmental domains, which is consistent with previous research results [16]. Elderly patients include features such as economic difficulties, poor mobility and lack of knowledge about tuberculosis compared to younger patients [37]. Moreover, some research has concluded that the peak age of diabetes mellitus and tuberculosis comorbidity was > 60 years [38–40]. Therefore, more psychological and social support and nursing interventions to increase the individual’s ability to manage everyday life should be provided on the basis of older TB characteristics. Our study also found that patients with comorbidities had lower scores in the physiological, social, and environmental domains, which is consistent with previous studies [41, 42]. Some studies suggest that both DR-TB and other comorbidities may interact to increase patient mortality and morbidity [41, 43, 44]. Currently, the management of this special population (e.g., elderly patients, comorbid diabetes, HIV) has become a focus of TB prevention and control efforts in China [45]. In the future, it is recommended that this issue strengthen research on such patients to continuously improve their QOL.

As mentioned in other reports, better QOL was found among higher income participants [12]. In our study, participants with lower family per capita monthly income had lower physiological and psychological domain scores. Unemployed patients reported poorer QOL in the physiological and social domains. Patients who were self-paying and living in rural areas had the lowest QOL scores in the physiological domain. The reason might be associated with the financial burden, and the ratio of the health expenditure of rural residents (5.87%~86.60%) was higher than that of urban residents (2.19%~32.25%) [46]. DR-TB patients have lost their jobs and have no source of income because of their poor health, which may indirectly affect the patient’s QOL [47, 48]. At the same time, the share of diagnostic expenditures and cure expenditures also continued to increase in these patients, and the reimbursement rate for second-line anti-TB drugs needed for treatment (linezolid, cycloserine, clofazimine) is relatively low [49, 50]. Health care costs imposed significant economic burdens on DR-TB patients and their families. Consequently, consultation services for treatment expenses are provided during the treatment and development of the policy with special health insurance.

### Limitations

Our study had several limitations. First, this was a cross-sectional study; hence, the ability to prove causation is limited. In the future, a prospective study needs to be conducted to confirm the findings of this study. Second, the included variables were assessed by self-report; thus, recall bias was unavoidable. Third, because the survey population was relatively small and limited to Nanjing, China, convenience sampling was used for this study. This method tends to result in a poorly representative sample with a high degree of chance, which ultimately has an impact on the results of the study, so it is not clear whether these results can be generalized to a larger population. Fourth, the internal consistency of the investigators was not adequately considered in this study, and uniform indicators should be used in future studies to evaluate the effectiveness of training after the investigators have been trained. Fifth, the QOL comparison was made with the national norm, the results of which were obtained among China’s general population in 2016. It would be more useful if a comparison with non-TB infected population in Nanjing were made. Yet, there isn’t any outcome of QOL among non-TB infected individuals in Nanjing by WHOQOL- BREF instrument. Finally, we did not examine the association between the QOL of DR-TB patients and important variables such as stigma, discrimination, and self-management.

## Conclusions

Our study revealed the present QOL of DR-TB patients in the Nanjing area. We found that the overall level of QOL among DR-TB patients was significantly low in Nanjing, and the environmental domains were the most salient. Some factors (e.g., age, residence, education level, family per-capita monthly income, payment method, course of the disease, ADRs, comorbidities, caregivers) may affect QOL. Consequently, there is a dire need to develop more effective and targeted interventions to improve QOL with respect to these factors.

### Electronic supplementary material

Below is the link to the electronic supplementary material.

Supplementary Material 1

## Data Availability

The datasets used and/or analyzed during the current study are available from the corresponding author upon reasonable request.

## Abbreviations

TB: Tuberculosis
QOL: Quality of life
DR-TB: Drug-resistant tuberculosis
WHO: World Health Organization
WHOQOL-BREF: World Health Organization Quality of Life-BREF
MTB: Mycobacterium tuberculosis
SD: Standard deviation
MR-TB: Mono-drug resistant tuberculosis
PDR-TB: Polydrug resistant tuberculosis
MDR-TB: Multi-drug resistant tuberculosis
XDR-TB: Extensively drug-resistant tuberculosis
ADRs: Adverse drug reactions
