# Supplementary material for: Assessing the quality of life in patients with drug-resistant tuberculosis: a cross-sectional study
Source: BMC Pulm Med. 2024 Jun 27;24:303. doi: 10.1186/s12890-024-03119-1 (PMC11210023; doi:10.1186/s12890-024-03119-1)
Supplement: Supplementary file 1 — Supplementary Material 1 [file 12890_2024_3119_MOESM1_ESM.docx]

**Table S1. Assignment of independent variables**

| Independent variable | Assignment Description |
| --- | --- |
| Gender | Male = 1,  Female = 2 |
| Age (year) | < 40 years old = 1,  40-60 years old = 2,  > 60 years old = 3 |
| Education level | Primary school or less = 1,  Junior High School = 2,  Senior School or Technical Secondary School = 3,  University or above = 4 |
| Marital Status | Married = 1,  Single = 2,  Divorced or widowed = 3 |
| Residence | Rural = 1,  Urban = 2 |
| Career | Unemployed = 1,  Farmer or worker = 2,  Staff = 3,  Students = 4,  Self-employed = 5,  Retirement = 6 |
| Family per-capita monthly income (RMB) | < 1500 = 1,  1500-3000 = 2,  3000-5000 = 3,  > 5000 = 4 |
| Payment method | Self-pay = 1,  Provincial or municipal medical insurance = 2,  New Rural Cooperative Medical = 3 |
| BMI | < 18.5 kg/m^2^ = 1,  18.5-22.9 kg/m^2^ = 2,  > 23.0 kg/m^2^ = 3 |
| Course of disease | < 1 month = 1,  1-6 months = 2,  6-12 months = 3,  > 12 months = 4 |
| ADRs | No = 1,  Yes = 2 |
| Comorbidities | No = 1,  Yes = 2 |
| Caregivers | No = 1,  Yes = 2 |

*Note.* ADRs, Adverse drug reactions
